# Acupuncture for the induction of labour: a double-blind randomised controlled study

**DOI:** 10.1111/j.1471-0528.2010.02647.x

**Published:** 2010-09

**Authors:** J Modlock, BB Nielsen, N Uldbjerg

**Affiliations:** a Department of Obstetrics and Gynaecology, Herning Regional HospitalHerning; b Department of Obstetrics and Gynaecology, Aarhus University HospitalSkejby, Denmark

**Keywords:** Acupuncture, labour induction, prolonged pregnancy, sham acupuncture

## Abstract

**Objective:**

To investigate whether acupuncture is effective for the induction of labour in post-term pregnancies.

**Design:**

A double-blind multicentre randomised controlled study.

**Settings:**

Aarhus University Hospital and Herning Regional Hospital, Denmark.

**Population:**

One hundred and twenty-five healthy women with uneventful pregnancies at gestational week 41^+6^ were randomised into two groups.

**Methods:**

The intervention group was given acupuncture twice on the same day at acupuncture point GV20 and bilaterally at points BL67, LI4 and SP6. The control group received sham acupuncture at the same points.

**Main outcome measures:**

At effect evaluation, which was carried out 24 hours after randomisation, the primary endpoint was labour or delivery.

**Results:**

The primary endpoint was achieved in seven women (12%) in the acupuncture group and eight women (14%) in the control group (*P* = 0.79). Stratification for parity and fetal gender did not alter the results.

**Conclusion:**

Under the treatment regimen investigated in this study, acupuncture for the induction of labour in post-term women at gestational age 41^+6^ weeks may not be effective.

## Introduction

Many pregnant women wish to take fewer medicines during pregnancy and labour, and this choice is often supported by midwives and obstetricians. The use of acupuncture is on the increase in labour wards despite limited scientific evidence of its effectiveness. We have been able to identify only four relatively small randomised controlled trials (RCTs) written in English describing the induction of labour by acupuncture. One of these RCTs (*n* = 25) demonstrated a positive effect of acupuncture, which induced cervical shortening after 6 days when given every other day starting at a gestational age of 40 weeks.^1^ The other three RCTs demonstrated no effect of acupuncture. Two of these RCTs included women with prelabour rupture of membranes (PROM), and determined time to delivery (*n* = 43)^2^ or time to the active phase of labour (*n* = 48).^3^ The remaining RCT included 181 post-term women, and found that the need for labour induction at gestational age 41^+3^ weeks was unchanged when acupuncture treatment was given on the two preceding days.^4^ It is well established that acupuncture is a safe procedure.^5^,^6^

Further RCTs investigating acupuncture for the induction of labour are needed for several reasons. Although there is an overall consensus in China regarding the location of the various energy points used for skin penetration,^7^ different types of acupuncture treatment are used in different traditions. Also, the number of women included in the above-mentioned RCTs was small. The objective of the present study was therefore to investigate whether acupuncture induces labour in pregnant women at gestational week 41^+6^, when given according to the guidelines generally used in Denmark and other European countries.^8^

## Methods

Ninety-five participants were enrolled in the study at Aarhus University Hospital, Skejby (approximately 4800 deliveries per year) from 1 December 2005, and 30 at the Herning Regional Hospital (approximately 2000 deliveries per year) from 1 February 2007. Enrolment ended on 31 May 2008. Both hospitals have neonatal departments on site.

Healthy pregnant women at gestational week 41^+6^ were eligible for participation in the study. They received written and oral information about the study, and those who agreed to participate gave written consent. Exclusion criteria were as follows: woman does not speak or understand the Danish language; multiple pregnancy; PROM or contractions at 4- to 5-minute intervals and increasing in intensity; previous caesarean section; diseases of the mother or unborn child (diabetes, pre-eclampsia, diseases of the heart, liver or kidneys, HIV/AIDS, malformation of the pelvis, psychological disorders, intrauterine growth restriction, hydrocephalus, suspected macrosomia, fetal malposition, antepartum stillbirth, treatment with anticoagulants, skin infections, allergy to metal, or major complications at previous delivery such as low Apgar score). Gestational ages were estimated using fetometric ultrasound parameters obtained before gestational week 20^+0^ weeks, or in 15 women using menstrual history.

Participants were randomised at a gestational age of 41^+6^ weeks immediately before the first acupuncture treatment, which was given at 8 a.m. Effect evaluations took place after 24 hours, and if necessary labour was induced on the same day using Dinoprostone® (at Herning), misoprostol (at Skejby) or amniotomy.

### Acupuncture procedure

In both groups the same fixed acupuncture points were used, and the needles appeared to be the same.^8^

- BL67. This point, it is claimed, can be used to stimulate uterine contractions; it is located on the little toe, near the edge of the nail, and the needle is inserted to a depth of 0.1–0.2 cm.^8^
- LI4. It is claimed that this point can be used to help the woman push downwards and to stimulate uterine contractions; it is located in the middle of interosseous I, and the needle is inserted to a depth of 1–2 cm.^8^
- SP6. Acupuncture at this point is claimed to promote ripening of the cervix; the point is located 3 cm above the medial malleoli, and the needle is inserted to a depth of 1.5–2.5 cm.^8^
- GV20. This point, it is claimed, can be used for calming; it is located 7 cun (11–14 cm) from the central part of the posterior hairline, and the needle is inserted to a depth of 0.5–1 cm.^1^,^8^

In the acupuncture group (AG), thin acupuncture Seirin B-type needles (Serin Corporation, Shizuoka, Japan) were used, whereas Park-Sham acupuncture needles were used in the control group (CG). In both groups, sticky tubes were used to conceal the type of needle used. The tube was fixed to the skin at the acupuncture point. The needle was then inserted into the tube. The real acupuncture needle penetrated the skin, while the sham needle had a blunt point so that the needle retracted into the needle handle and did not penetrate the skin.^9^,^10^ All the midwives were trained in acupuncture according to the guidelines described by Deadman *et al.*,^8^ and they were all regular practitioners of acupuncture, performing acupuncture treatments approximately five to six times a week.

After cardiotocographic monitoring and randomisation, women were given the treatment, which began at approximately 8 a.m. The treatment lasted 30 minutes, and during this time the needle points were stimulated by manual twirling of the needles every 10 minutes. If the primary endpoint had not occurred, the treatment was repeated at 2:30 p.m. without a preceding cardiotocograph.

### Outcomes

At effect evaluation 24 hours after the first acupuncture treatment, the primary outcome was achieved if the participant had undergone delivery or was in active labour, defined as rupture of fetal membranes and/or contractions at 4- to 5-minute (or more frequent) intervals and increasing in intensity. Observations were noted on a form on which all outcomes of interest with regard to effect evaluation were recorded.

Secondary outcomes were as follows: the cervical dilatation was sufficient for amniotomy, cervical length and dilatation, length of labour, time from randomisation to start of active labour, postpartum bleeding, use of epidural, augmentation of contractions and instrumental delivery, as well as neonatal outcomes such as Apgar score and umbilical pH value when available. All secondary endpoints were obtained from the files of the randomised participants and noted on a research form.

### Randomisation

Treatments were allocated by the acupuncturist using a computer-randomisation system accessible through an ordinary telephone line (voice response). After entering the woman’s unique personal identification number and current parity (nulliparous/parous), treatments were allocated using block randomisation with stratification for parity. The randomisation system complied with international criteria for proper concealment of randomisation.^11^,^12^

The acupuncturists were not blinded to which treatment was given, but the midwives who performed the effect evaluations and the principal investigator who gathered the data and conducted the telephone interviews were blinded. In the follow-up telephone interview, the women who were contacted (63%) were asked whether they knew to which group they had been allocated. If it was not possible to reach a woman after two calls, no further attempts at contact were made.

### Statistical analysis

Accepting a 5% risk of type 1 error (alpha) and a 20% risk of type 2 error (beta), and by assuming that the primary outcome was achieved in 10% of the CG^13^ and in 30% of the AG, it was calculated that 62 participants were needed in each randomised group.

Statistical analysis was performed using the software program Stata/IC 10 (Stata Corporation, College Station, TX, USA). All data were analysed according to the intention-to-treat principle. Analysis of all proportions was carried out using two-sided Fisher’s exact test and Pearson’s chi-square test when appropriate. Two-sample Wilcoxon rank-sum tests were used to compare medians for unevenly distributed observations such as postpartum bleeding and cervical dilatation. All outcomes, where relevant, were stratified for parity.

## Results

**Figure 1:**
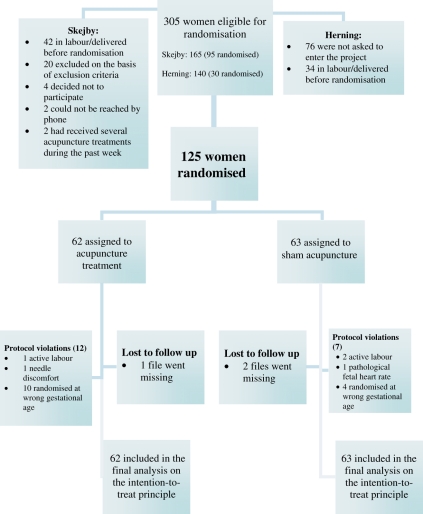
CONSORT flow diagram.

In total, 305 eligible women were invited to participate in the study, and 125 pregnant women were randomised: 30 women from Herning and 95 from Skejby. Nineteen of the women randomised violated the protocol (Figure 1). Three files disappeared, and for these women, it was not possible to retrieve the data (Figure 1).

Most probably, approximately 970 women were eligible for the study as the post-term rate at the two hospitals was 8.9%. However, only 305 were invited to participate as a result of the apparent forgetfulness of the midwives. There is no evidence that women who were eligible to participate but not invited differed from those who were invited to do so.

After randomising one woman in the early stages of the study period who had had acupuncture within the past week before randomisation, we decided for the remainder of the study period to exclude all women who had recently received similar treatment. This was to avoid any possible effect of this earlier treatment. Because in two women at the beginning of the study period their partners saw the needles and thereby identified the allocation, we decided that women could not have a partner or relative present during the needle session.

There was some difficulty in getting the tube to adhere to the skin at acupuncture point BL67. We therefore decided to omit the tube at this point when treating the AG. There was no report of any adverse event as a result of the acupuncture treatment.

No differences were observed between the AG and the CG in terms of age, gestational age, parity, body mass index, use of tobacco, birthweight or head circumference (Table 1). The blinding failed in six instances: four women had their treatment effect evaluated by the same midwife who conducted their randomisation, and two women were informed about their allocation by their partners; these failures were evenly distributed between the two groups. However, as demonstrated in Table 2, the blinding worked satisfactorily as most of the women did not know what type of treatment they had received.

**Table 2:** Blinding

Actual allocation	Group to which participants believed they were allocated						
	Control group		Acupuncture group		No idea		*P* value
Acupuncture group (*n*=39): *n*; % (95% CI)	5	13% (4–27)	8	10% (9–37)	26	66% (50–81)	0.36*
Control group (*n*=40): *n*; % (95% CI)	7	18% (7–33)	5	13% (4–27)	28	70% (54–83)	

* Pearson’s chi square test.

In a follow-up telephone interview, the participants were asked whether they knew to which group they had been allocated.

**Table 1:** Demographic data

	Acupuncture group (95% CI)		Control group (95% CI)	
**Maternal data**				
No.	62		63	
Age (years): (mean (95% CI)	31 (30–32)		30 (29–32)	
Nulliparous: *n*; % (95% CI)	36	58% (45–75)	38	60% (47–72)
BMI >30: *n*; % (95% CI)	7	12% (5–22)	3	5% (1–13)
BMI <20: *n*; % (95% CI)	7	12% (5–22)	6	11% (4–20)
Tobacco use: *n*; % (95% CI)	8	14% (6–24)	3	5% (1–13)
**Neonatal data**				
Male/newborns: *n*/*n*; % (95% CI)	28/60	47% (34–60)	36/57	63% (49–76)
Birth weight (kg): mean (95% CI)^*n*^	3897	(3.765–4.029)^60^	3914	(3.799–4.030)^58^
Head circumference (cm): mean (95% CI)^*n*^	36.0	(35.6–36.4)^59^	36.1	(35.8–36.5)^56^

There was no significant difference (relative risk 0.85; 95% CI 0.33–2.2) between the groups with regard to primary outcome, which was the number of women who went into active labour or underwent delivery within 24 hours (Table 3). In addition, there were no significant differences in secondary outcomes between the groups (Table 3). After stratification for parity and re-analysis of the data following exclusion of 19 participants who violated the protocol, this absence of effect of acupuncture remained.

**Table 3:** Effect evaluation 24 hours after randomisation

	**Acupuncture group (95% CI)**		**Control group (95% CI)**		***P*-value**
**Delivered/in labour (primary outcome)**					
Total: *n*/*n*; % (95% CI)	7/60*	12% (5–23)	8/58*	14% (6–25)	0.79**
Nulliparous	3/36	8% (2–22)	3/35	9% (2–23)	1.00**
Parous	4/24	14% (5–37)	5/23	23% (8–44)	0.72**
Cervical maturity***					
Cervical length <1 cm: *n*/*n*; % (95% CI)	28/55	51% (37–65)	33/51	65% (50–78)	0.17**
Cervix dilatation (cm): mean; SD^*n*^	1.5	0.86^56^	1.1	0.77^52^	0.90****
Amniotomy possible: *n*/*n*; % (95% CI)	16/60	27% (16–40)	19/58	33% (21–46)	0.55**
Nulliparous	7/36	19% (8–36)	7/35	20% (8–37)	1.00**
Parous	9/24	38% (19–59)	12/23	52% (31–73)	0.39**
**Epidural**					
Epidural: *n*/*n*; % (95% CI)	24/60	40% (28–53)	24/57	42% (29–56)	0.85**
Nulliparous	19/36	53% (36–70)	20/34	59% (41–75)	0.64**
Parous	5/24	21% (7–42)	4/23	17% (5–39)	1.00**
**Length of labour**					
Total (minutes): mean (95% CI)	448 (374–522)		403 (333–474)		0.38*****
First stage	390 (319–461)		357 (290–425)		0.51*****
Second stage	41 (30–52)		60 (2–118)		0.52*****
**Postpartum bleeding**					
Postpartum bleeding >500 ml:*n*/*n*; % (95% CI)	15/59	25% (15–38)	13/55	24% (13–37)	1.00**
**Stimulation of contractions**					
Total: *n*/*n*; % (95% CI)	26/47	55% (40–70)	22/39	56% (40–72)	1.00**
Primiparous	19/28	68% (48–84)	17/21	81% (58–95)	0.35**
Parous	7/19	37% (16–62)	5/18	28% (10–53)	0.73**
**Instrumental delivery**					
No intervention: *n*; % (95% CI)	41	68% (57–80)	39	67% (55–79)	0.78******
Ventouse: *n*; % (95% CI)	8	13% (5–22)	8	14% (5–23)	
Caesarean section: *n*; % (95% CI)	11	18% (9–28)	11	19% (9–29)	
**Neonatal outcome**					
Apgar ≤ 7					
1 minute: *n*/*n*; % (95% CI)	8/60	13% (6–25)	6/58	10% (4–21)	0.78**
5 minutes: *n*/*n*; % (95% CI)	1/60	2% (0–9)	1/58	2% (0–9)	1.00**
**Umbilical pH value**					
<7.2: *n*/*n*; % (95% CI)	13/44	30% (17–45)	15/48	31% (19–46)	1.00**
<7.1: *n*/*n*; % (95% CI)	2/44	5% (1–16)	3/48	6% (1–17)	1.00**

* The difference between the total numbers of inclusions in the AG and CG (62 and 63 respectively) and the numbers analysed in each of the two groups reflect inconsistencies in reporting.

** Two-sided Fisher’s exact test (intention-to-treat principle).

*** Includes all women who had not yet undergone delivery, including women in active labour.

**** Two-sample Wilcoxon rank-sum test.

***** Two-sample *t* test.

****** Pearson’s chi-square test.

## Discussion

The results of this randomised controlled study revealed no effect of acupuncture on induction of labour in post-term women. Neither the primary endpoint (labour or delivery after 24 hours) nor the secondary endpoints differed between the groups. The risk of a clinically significant type 2 error is low because the primary endpoint was achieved in 12% of the acupuncture group compared with 14% in the control group, with a 95% CI of 0.33–2.2 for the relative risk.

The strengths of this study are that international standards were adhered to in terms of blinding (which was based on sham needles) and allocation concealment, in contrast to the three RCTs detailed in Table 4. Furthermore, an analysis of the success of blinding was performed, which indicated that the technical problems we had with acupuncture point BL67 and the possible prior experience of some participants with acupuncture did not affect the blinding. The limitations of this study include 19 violations of the protocol. This is a relatively large number; however, the violations were evenly distributed between the groups. pH values were obtained from only 92 neonates, primarily those at Skejby because, at the time of the study, umbilical blood tests were only performed at Herning if Apgar scores were low. Therefore, this secondary endpoint should be interpreted with caution. Sham acupuncture may have an effect beyond that of placebo when used to treat pain in nonpregnant women^14^). Such an effect would cause an underestimation of the effect of acupuncture on the induction of labour.

**Table 4:** Randomised controlled trials on acupuncture for the induction of labour

Inclusion criteria	Endpoint	*n*	AG	CG	*P* value
Post-term (this study)	Need for induction 24 hours after treatment	125	88%	86%	0.46
PROM^3^	Time to active phase of labour	106	15 hours	21 hours	0.34
PROM^2^	Need for induction 24 hours after PROM	100	35%	40%	0.64
Term^1^	Need for induction after treatment every second day from EDC	46	20%	35%	0.30
Post-term^4^	Need for induction 3 days after treatment	364	72%	69%	0.83

EDC, estimated day of confinement.

In Table 4, we have listed five RCTs investigating the effect of acupuncture on the induction of labour. The four largest of these RCTs did not demonstrate any effect, which suggests that the acupuncture regimes used in these trials may not be effective for the induction of labour.

Many acupuncturists argue that to achieve the optimal effect of acupuncture, it is important that the treatment be customised according to the diagnostic process that is integral to the traditional Chinese system of medicine. The women included in a study by Selmer-Olsen *et al.* were treated according to this philosophy and were categorised into three traditional Chinese medicine groups. However, the results did not indicate any difference in the effect of acupuncture among the groups.^3^ It could also be argued that, in our study, a more intensive course of treatment in the post-term women may have produced an effect on the outcome parameters.

The subsequent telephone interviews suggested that participation in this study appeared to have been a positive experience for the women. They were eager to help search for alternatives to the conventional methods of induction. They also found that spending a day in hospital was a welcome distraction during the difficult period of waiting for labour to begin, particularly as they were doing something that made sense to them. However, as acupuncture does not seem to have an effect on the induction of labour, alternative distractions must be sought.
